# Cases Journal: time for a new path

**DOI:** 10.1186/1757-1626-2-9122

**Published:** 2009-12-01

**Authors:** Richard Smith

**Affiliations:** 1 BioMed Central, Floor 6, 236 Gray's Inn Road, London, WC1X 8HL, UK

## 

Help us find a new way to achieve our aims.

The aim of *Cases Journal *is to publish tens of thousands of reports of "ordinary" cases. We want to do this to illustrate how no case is normal, to give doctors and patients a chance to reflect on cases through writing about them, to provide material for learning, and eventually to build a database that will be useful to doctors and patients alike.

So far we have succeeded in publishing hundreds of cases but not tens of thousands. It's time to try a different tack, and the transfer of *Cases Journal *and the *Journal of Medical Case Reports *to BioMed Central gives us a chance to do that.

The *Journal of Medical Case Reports*, our sister journal, will continue progressing along a similar path to now -- publishing original and important case reports, developing the science of case reports, and steadily increasing their academic value.

*Cases Journal *will, in contrast, try to find a new path. The current journal will cease publishing, but everything that has been published will remain archived, easily found, and open access. Meanwhile, we'll try to find a new path, and we are interested in all ideas.

When metamorphosed Cases will almost certainly cease to be a journal. We tried the journal model initially because we thought that people wanted to publish in journals -- even though it's an increasingly archaic and cumbersome form that requires expensive apparatus. Academics are showing themselves willing to write blogs and publish on "databases" like *PLoS One, PloS Currents*, and *Nature Communications*, and many academics contribute more to social networking sites like Facebook and Twitter than they do to journals. And we are interested not only in academics but also in ordinary clinicians. These clinicians don't need the imprimatur of a journal but simply need an easy way to share their stories.

One thing I've discovered through teaching a course on writing and publishing cases is that many doctors, particularly older ones, find it very refreshing to write case reports that are not the classical case report of a surgical rarity but a much more human story of how doctors partner with patients to help them through their problems. Some of these stories provide deep insights into what it is to be a doctor and what the doctor patient relationship really means to both parties, material that is not available in traditional case reports.

These stories were often written with the patients and provide startling insights into the different perspectives of doctors and patients. One doctor described his near despair at his inability to help a grossly obese patient with a very unhealthy lifestyle, severe asthma, and a violent disposition. The doctor and patient had been meeting for years, and the doctor began his case report with a consultation where he felt completely hopeless and wondered what it meant to be a doctor and so powerless. Yet the patient saw that consultation as a turning point. He adopted a healthier lifestyle and lost weight and both his asthma and his violent disposition improved.

So at least some ordinary doctors want to write to reflect on their practice and to share their learning. But some patients may be equally motivated to tell their stories, both to make sense of them themselves and to share the learning from their case histories. With our journal mentality we wanted cases from doctors, but now we are interested in collecting cases from patients and doctors--and preferably from both at the same time.

We would like to create a space where patients and clinicians can create, share, and learn together, something that could be immensely valuable in advancing the important agenda of making the clinician-patient partnership a much more equal one.

We welcome ideas on how we can find a new path, and we look forward to our return as a butterfly and no longer a caterpillar.

